# Paediatric perioperative hypersensitivity reactions: Analysis of the French GERAP network database

**DOI:** 10.1111/pai.70474

**Published:** 2026-09-16

**Authors:** Mertes Paul Michel, Pouessel Guillaume, Gouel Aurélie, Karila Chantal, Lejus‐Bourdeau Corinne, Franchina Sébastien, Tacquard Charles, For the GERAP Network, Perquin Mélanie, Husser Solenne, Morisset Martine, Huynh Vinh An, Charansol Angéline, Hanniet Anatole, Florence Lakkis‐Castelain, Bordes Demolis Maryline, Giraudon Antoine, Pellerin Christelle, Vaia Elleni, Mariotte Delphine, Mure Marion, Ollivier Yann, Serrier Julien, Dalampira Georgia, Baud Charlotte, Muti Daniela, Schoeller Estelle, Capo‐Chichi Rosita, Seltzer Sandrine, Vandenberghe Durr Sophie, Pottier Eloïse, Zambelli Valentina, Mear Amélie, Facon Alain, Ledesve D'heudieres Pierre, Pelletier De Chambure Diane, Caron Juliette, Delebarre‐Sauvage Christine, Pouessel Guillaume, Bellet Elisabeth, Orsel Isabelle, Le Quang Diane, Tezier Marie, Boghossian Marie‐Caroline, Fargeas Marine, Fresco Raphaëlle, Gouitaa Marion, Billard Carine, Lefevre Sébastien, Chiriac Anca, Demoly Pascal, Fayard Blandine, Beaugendre Isabelle, Colas Luc, El Hanache, Aguinet Emmanuelle, Petit Isabelle, Rezzadori Gilles, Pallancher Stéphanie, Miran Sophie, Patel Minaxi, Merzouk Aicha, Smilov Magdalena, Barakat Leyla, Dechaisemartin Luc, Gouel‐Cheron Aurélie, Neukirch Catherine, Martin Aurélie, Merlin Karine, Bailleul Amaury, Demiri Migena, Fessenmeyer Christine, Karila‐Beaulier Chantal, Lepage David, Gest Noémie, Seringulian Alice, Taing Diane, Hamon Gautier, Verliere Ambre, Saf Sarah, Lequipe Johan, Renauld Valérie, Malinovsky Jean‐Marc, Dessard Sabrina, Le Guillou Lisa, Rochefort‐Morel Cecile, Franchina Sébastien, Girard Emmanuel, Nafeh Samer, Delzanno Cédric, Dzviga Charles, Million‐Charrel Justine, Minguy Nolwenn, Tacquard Charles, Viville Simon, Le Guen Morgan, Gayraud Jacques, Gil Céline, Maihol Claire, Migueres Isabelle, Chassery Clément, Herry Julie, Hoarau Cyrille, Barakat Leyla, Faivre Sébastien

**Affiliations:** ^1^ UMR_S1255 EFS‐INSERM Unit Strasbourg France; ^2^ Department of Paediatrics Children's Hospital Roubaix France; ^3^ Paediatric Pulmonology and Allergy Department Jeanne de Flandre Hospital, Lille University Hospital (CHU) Lille France; ^4^ Lille University, ULR 2694: METRICS Lille France; ^5^ University Paris Cité Paris France; ^6^ Anesthesiology and Critical Care Medicine Department DMU PARABOL, Bichat Hospital, AP‐HP Paris France; ^7^ Antibody in Therapy and Pathology, Pasteur Institute, UMR 1222 INSERM Paris France; ^8^ Paediatric Pulmonology and Allergy Department Necker‐Enfants Malades Hospital, Paris‐Descartes University Paris France; ^9^ Department of Anaesthesia and Intensive Care CHU Nantes, Hôtel Dieu ‐ Hôpital Mère Enfant Nantes France; ^10^ Perioperative Allergic Reaction Exploration Unit, Department of Anesthesia and Intensive Care Rouen University Hospital Rouen France; ^11^ Department of Anaesthesia and Intensive Care Strasbourg University Hospital Strasbourg France; ^12^ UMR_S1255 EFS‐INSERM Unit, EFS Grand Est Strasbourg France

**Keywords:** anaphylaxis, children, neuromuscular blocking agents, perioperative hypersensitivity, tryptase

## Abstract

**Background:**

Perioperative hypersensitivity (POH) reactions are rare but potentially life‐threatening and remain poorly characterised in children. We analysed the largest available French paediatric cohort to describe the epidemiology, causal agents, temporal trends and phenotypes.

**Methods:**

We pooled data from paediatric patients (<18 years) included in six previously published GERAP surveys (1997–2012) and the GERAP computerised database (2017–2024), all evaluated in centres of the GERAP network according to current guidelines. The primary objective was to compare POH phenotypes with and without causal agent identification.

**Results:**

Of 4605 POH reactions recorded, 461 (10%) occurred in children (median age 14 [10–16] years; 47% female). Reactions were grade 1 in 50%, grade 2 in 23%, grade 3 in 26% and grade 4 in 1%. The causal agent was identified in 199 (43%) cases, mainly neuromuscular blocking agents (NMBAs) (31%), latex (30%) and antibiotics (16%). Identification was more frequent in anaphylaxis than in isolated cutaneous reactions (61% vs. 25%, *p* < .001). Latex‐related reactions disappeared after 2017. Patients in whom a causal agent was identified had more severe reactions (grade 3–4: 46% vs. 13%; *p* < .001), more cardiovascular and respiratory involvement, and higher acute serum tryptase (median 16.0 vs. 4.0 μg/L; *p* < .001) than those without identification.

**Conclusions:**

Paediatric POH are not uncommon and may be life‐threatening. All suspected reactions, including isolated cutaneous signs, warrant referral to specialised allergy centres given the still low identification rate and the continuing burden of NMBA‐ and antibiotic‐related reactions.

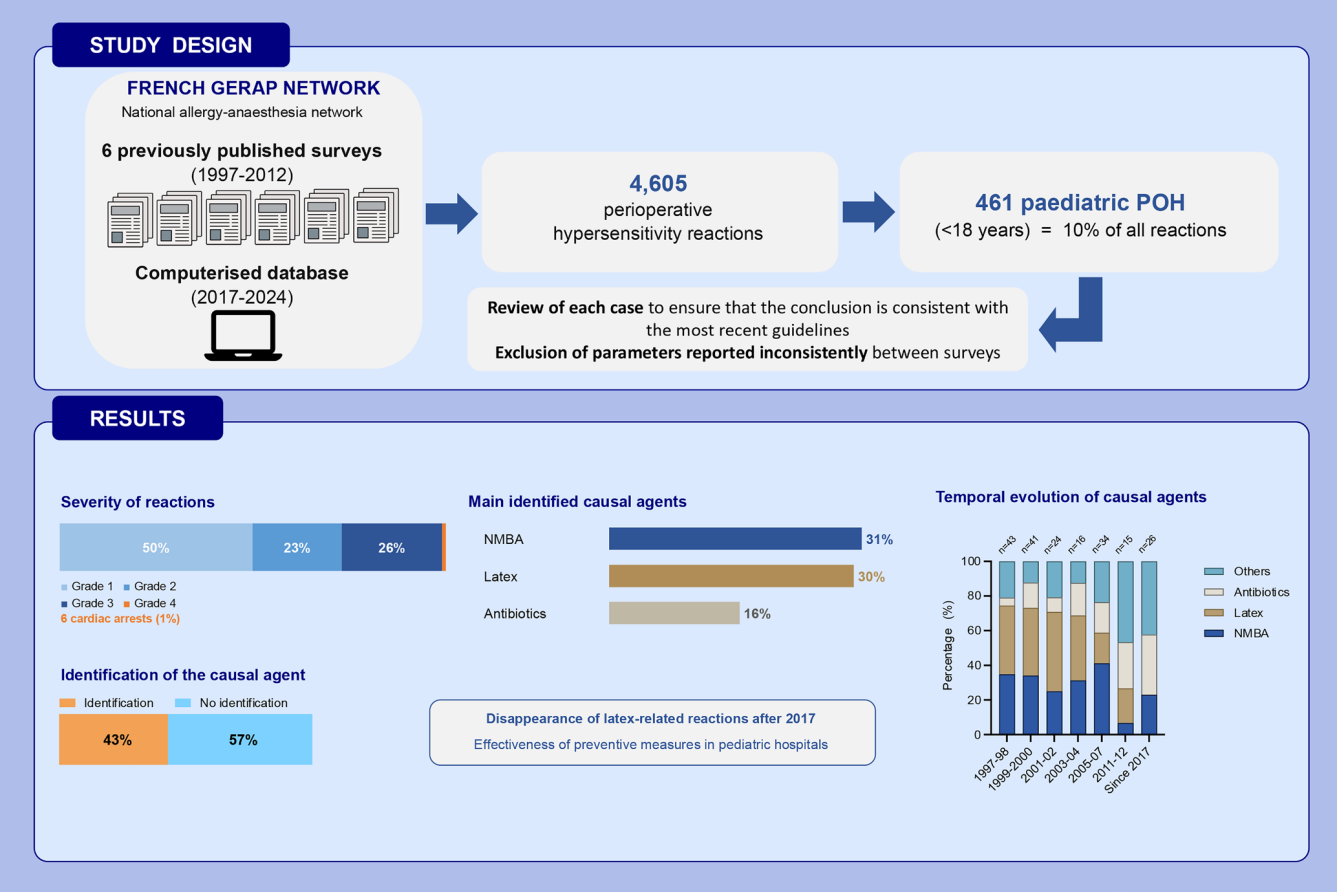

## Graphical abstract

Graphical abstract of the pediatric perioperative hypersensitivity reactions recorded in the GERAP network datbase between 1997 and 2024.

**Key message**

Paediatric perioperative hypersensitivity reactions account for 10% of all recorded reactions. They are generally less severe, although some can be life‐threatening. The main causal agents were neuromuscular blocking agents, latex and antibiotics, but latex‐related reactions disappeared after 2017.

## 1 INTRODUCTION

Immediate perioperative hypersensitivity reactions (POH) are rare but with potentially life‐threatening complications, posing significant diagnostic and therapeutic challenges for anaesthetists and allergists. While these reactions have been extensively documented in adults,^1^ little attention has been provided to the paediatric aspect of this complication in the literature, despite potential differences in epidemiology, causal agents and clinical presentation between children and adults.

The incidence of POH in children remains poorly reported, with significant variation across geographic regions and methodological approaches. The APRICOT study, a large European multicentre investigation involving 30,874 paediatric anaesthetic cases, identified only three cases of anaphylaxis, yielding an estimated incidence of 1 in 10,000,^2^ close to that reported in adults.^3^ Other national reports suggest a lower incidence rate, such as in the Sixth National Audit Project (NAP6) in the United Kingdom, with an incidence of severe anaphylaxis (grade 3 and 4 according to Ring and Messmer classification) of 2.7 per 100,000 paediatric anaesthetic procedures.^4^ In the United States of America (USA), the incidence has been estimated between 1 in 12,125 and 1 in 36,479 anaesthetic procedures.^5^, ^6^ In Japan, the incidence of POH in children was estimated at 0.018%, compared to 0.03% in Turkey and 0.04% in Singapore.^7^, ^8^, ^9^ All previously published studies on paediatric POH have relied either on small cohorts or on large nationwide de‐identified datasets with serious methodological shortcomings, both of which limit the ability to characterise the associated phenotype and the allergy workup.

In France, the epidemiology of POH has been systematically monitored since 1989 by the ‘Groupe d'Etude des Réactions Anaphylactiques Periopératoires’ (GERAP), a national network of allergy centres bringing together allergists, anaesthesiologists and biologists, dedicated to the investigation and data collection of these reactions. Since its establishment, the GERAP has conducted 11 published serial epidemiological surveys that have substantially advanced understanding of the evolving patterns of POH, but mainly in adults.

However, a thorough understanding of the specific characteristics of POH in children is essential to optimise prevention strategies, improve diagnostic accuracy and ensure safer anaesthetic practices in this vulnerable population.

Based on the GERAP expertise, we aimed to conduct an analysis of all paediatric cases of POH from 1997 to 2024, in order to describe the epidemiology, time trends according to the causal agents and the phenotypes according to the identification or not of the causal agent.

## 2 METHODS

### 2.1 Eligibility criteria and ethics approval

**FIGURE 1:**
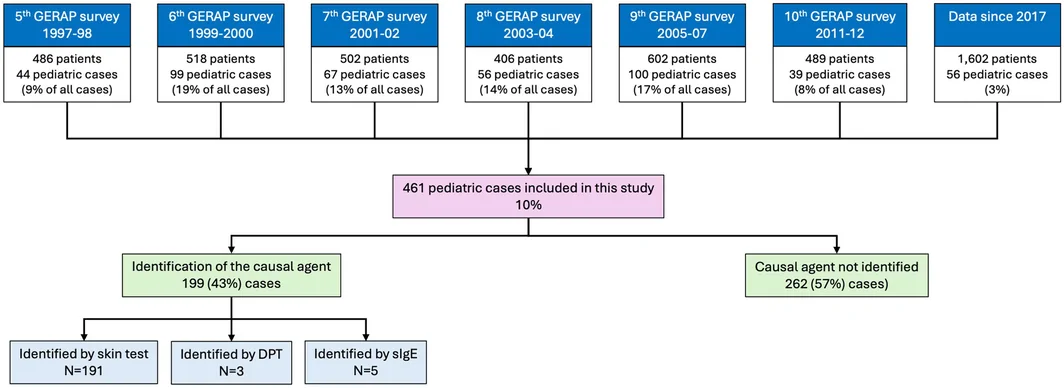
FlowChart of the study. DPT, Drug provocation test; sIgE, Specific immunoglobulin E. The percentages shown in the first row of the flowchart represent the percentage of paediatric cases among all cases in the same survey.

This study was based on a new analysis of raw data from paediatric patients (<18 years of age) included in the six previously published GERAP surveys that combined both adult and paediatric population [5th:1997–1998^10^; 6th: 1999–2000^11^; 7th: 2001–2002^12^; 8th: 2003–2004^13^; 9th: 2005–2007^14^; 10th: 2011–2012^15^], as well as data recorded in the computerised database from 2017 to February 2024 (Figure 1). Some patients extracted from the database after 2017 were already described in the survey published in 2025, which covered 2017 and 2018.^1^

Patients were included in this study if they experienced a POH reaction and were evaluated at a GERAP network allergy centre. Only patients with a confirmed diagnosis of POH after allergy testing were included in the GERAP database.

Ethical approval for patients from previously published studies was described in the respective publications and was in accordance with French legislation at the time of publication. For patients recruited after 2017, the study was approved by the Strasbourg institutional ethics committee (CE‐2020‐189) and registered within ClinicalTrials.gov (NCT04654923). Informed consent was obtained from their legal representative. The study was conducted in accordance with the principles of the Declaration of Helsinki.

### 2.2 Data collection

The recorded data included: patient's characteristics, medical history, conduct of anaesthesia, clinical signs (phenotype) of the POH and results of the allergy workup. Skin prick and intradermal tests were carried out for at least all drugs administered during the anaesthetic procedure and recorded on the anaesthetic chart, as well as for latex. Specific IgE (sIgE) to quaternary ammonium ions and to latex, and acute serum tryptase and plasma histamine sampled at the time of the reaction (ideally 30 min–2 h after onset), were assessed when available. The severity of each reaction was graded from 1 to 4 according to the modified Ring and Messmer classification. Tryptase was measured by fluoroenzyme immunoassay (ImmunoCAP, Thermo Fisher Scientific; upper reference limit 11.4 μg/L during the study period) and sIgE by ImmunoCAP (positivity ≥0.35 kUA/L). Drug provocation tests were performed selectively and not routinely for neuromuscular blocking agents (NMBAs) or radiocontrast media. Chlorhexidine was not part of the systematic testing panel during the first part of the study period, and basophil activation testing was not available.

### 2.3 Conduct of the study

The database of paediatric patients from each of the previously published GERAP surveys was merged with the most recent data issued from the computerised database. The data availability according to source is described in Table S1. Variables that were not recorded consistently across the successive surveys were not included in the corresponding analyses (e.g. basal serum tryptase, encoded only in the database since 2017); no patient was excluded on this basis. The allergy workup was conducted according to the up‐to‐date guidelines at the time of the reaction: the 2001 and 2011 guidelines from the French Society of Anesthesia and Intensive Care (SFAR), and the 2019 guidelines from the European Academy of Allergy and Clinical Immunology (EAACI).^16^, ^17^, ^18^

To enable comparison between studies, the conclusions were harmonised based on the interpretation of skin test results, sIgE and drug provocation tests and all records were individually reviewed by CT. Skin tests that were positive only at concentrations now considered irritant according to the EAACI recommendations (for example intradermal tests to NMBA at concentrations exceeding the recommended non‐irritating maximal concentration, such as atracurium above a 1:1000 dilution) were therefore reinterpreted as negative.^18^

### 2.4 Study endpoint

The primary objective was to compare the POH phenotypes with and without identification of the causal agent. The secondary objectives included describing the causal agents by time trends and according to age groups and severity of the reaction.

### 2.5 Statistical analysis

Quantitative data were expressed as the median and interquartile range [IQR], while qualitative data were expressed as the number and proportion. *p*‐values were calculated using the Mann–Whitney test or Pearson's chi‐squared test for categorical comparisons and comparisons of the evolution over time. All of these tests were performed using Prism 10 software (GraphPad, San Diego, USA). A 95% confidence interval was calculated using Wilson/Brown formula.

## 3 RESULTS

### 3.1 Main characteristics of the POH reactions

Between 1997 and 2024, 4605 POH reactions were included in the GERAP database, with 461 (10.0%) cases of paediatric POH identified (Figure 1). The median age was 14 (IQR: 10–16; range: 1–17) years, and 216 patients (47%) were female (Supplementary Figure S1). Data regarding the type of anaesthesia were available in 311 patients. Most reactions occurred during general anaesthesia (*n* = 284; 91% of patients with available data), primarily during anaesthetic induction (*n* = 184; 66% of patients with available data). Table 1 shows the main characteristics of the cohort.

**TABLE 1:** General characteristics of the paediatric population of the GERAP database.

Variables	Results
**General characteristics**	
Sex Female	216 (47%)
Age (years)	14 [10–16]
**Medical history**	
Atopy	145 (31%)
Asthma	72 (16%)
Drug allergy	66 (14%)
Food allergy	35 (8%)
Latex allergy	73 (16%)
Previous POH	7 (2%)
Chronic medication with betablockers	2 (0%)
Previous anaesthesia	248 (54%)
**Type of anaesthesia**	
General anaesthesia	284 (62%)
General anaesthesia + local regional anaesthesia	7 (2%)
Local regional anaesthesia	9 (2%)
Local anaesthesia	11 (2%)
Unknown	150 (33%)
**Timing of the reaction**	
Induction	184 (40%)
Maintenance	49 (11%)
Recovery room	44 (10%)
Unknown	184 (40%)
**Severity grade**	
Grade 1	229 (50%)
Grade 2	106 (23%)
Grade 3	120 (26%)
Grade 4	6 (1%)
**Description of the reaction**	
Cutaneous signs	376 (82%)
Erythema	275 (60%)
Urticaria	117 (25%)
Oedema	59 (13%)
Cardiovascular signs	138 (30%)
Tachycardia	64 (14%)
Bradycardia	5 (1%)
Hypotension	68 (15%)
Cardiovascular collapse	77 (17%)
Cardiac arrest	6 (1%)
Respiratory signs	133 (29%)
Bronchospasm	129 (28%)
Hypoxemia	17 (4%)

*Note*: Data are presented as *n* (%) and median [IQR]. POH: Perioperative hypersensitivity reaction. Atopy was defined as a personal history of at least one physician‐diagnosed atopic disease (allergic asthma, allergic rhinoconjunctivitis, atopic dermatitis and/or IgE‐mediated food allergy).

Most of these reactions were not severe, with 229 patients (50%) showing a non‐anaphylactic grade I reaction. The other 232 patients experienced anaphylaxis, including 106 (23%) grade 2 reactions, 120 (26%) grade 3 reactions and six (1%) cases of cardiac arrest. The POH phenotypes are shown in Table 1. After the reaction, surgery was continued in 172 (90%) of the 192 patients for whom this information was available and was discontinued in the remaining 20 (10%). Among these 20 patients, 14 (70%) had experienced severe anaphylaxis (grade 3–4).

### 3.2 Results of the allergy workup

The median time‐frame between the initial reaction and the allergy workup was 4 months (IQR: 2–9). Acute serum tryptase and histamine results were available for 154 (33%) and 66 (14%) patients, respectively. The median acute serum tryptase value was 6.5 [3.0–17.3] μg/L, and the median acute histamine value was 12 [IQR: 4–100] nmol/L. Acute serum tryptase exceeded 11.4 μg/L in 57 (37%) patients. Only 28 (6%) patients had basal serum tryptase value available, which precluded its use in defining mast cell activation in our cohort. Latex‐ and quaternary ammonium‐sIgE were collected from 94 (20%) and 148 (32%) patients, respectively, and were positive (>0.35 kUI.L‐1) in 50 (53%) and 36 (24%) cases, respectively. Skin tests for any of the suspected causal agents were positive in 191 patients (41%, range of the age 2–17). Three patients had positive drug provocation tests (acetaminophen, *n* = 2; ceftriaxone, *n* = 1) and 5 had isolated sIgE (latex, *n* = 4; quaternary ammoniums, *n* = 1). None of the patients had a basophil activation test result available. Based on a concordant clinical history, positive skin tests, and/or positive sIgE and/or positive challenge test, the causal agent was identified in 199 cases (43%) (Table 2, Table S2).

**TABLE 2:** Causal agent identified after the allergy workup.

Identification of the causal agent	*n* = 199	Type of agent identified
Neuromuscular blocking agents	61 (31%)	Atracurium: 20 (10%) Suxamethonium: 12 (6%) Rocuronium: 11 (6%) Mivacurium: 10 (5%) Vecuronium: 6 (3%); Mivacurium and suxamethonium: 1 (1%) Cisatracurium: 1 (1%)
Latex	59 (30%)	
Antibiotics	32 (16%)	Vancomycin: 11 (6%) Amoxicillin ± clavulanic acid: 9 (5%) Cefazolin: 6 (3%) Cefuroxime: 2 (1%) Ceftriaxone: 2 (1%); Ofloxacin: 1 (1%) Other^a^: 1 (1%)
Hypnotics	14 (7%)	Ketamine: 5 (3%) Midazolam: 4 (2%) Propofol: 4 (2%) Thiopental: 1 (1%)
Opioids	7 (3%)	Morphine: 3 (2%) Sufentanil: 2 (1%) Fentanyl: 1 (1%) Nalbuphine: 1 (1%)
Colloids	5 (2%)	Hydroxyethyl starch: 3 (2%) Gelatins: 2 (1%)
Local anaesthetic	2 (1%)	Lidocaine: 1 (1%) Bupivacaine: 1 (1%)
Other	19 (10%)	Nefopam: 3 (2%) Acetaminophen: 3 (1%) Dexamethasone: 3 (1%) Aprotinine: 2 (1%) Nefopam and morphine 1 (1%) Propacetamol: 1 (1%) Methylprednisolone: 1 (1%) Gadolinium: 1 (1%) Hydroxyzine: 1 (1%) Tramadol: 1 (1%) Sugammadex: 1 (1%) Chlorhexidine: 1 (1%)

*Note*: Results are expressed as *n* (%).

^a^ Other: Oone case in which an antibiotic was identified as the culprit but the specific molecule could not be retrieved from the record.

### 3.3 Causal agents and distribution by time periods, age groups and severity

**FIGURE 2:**
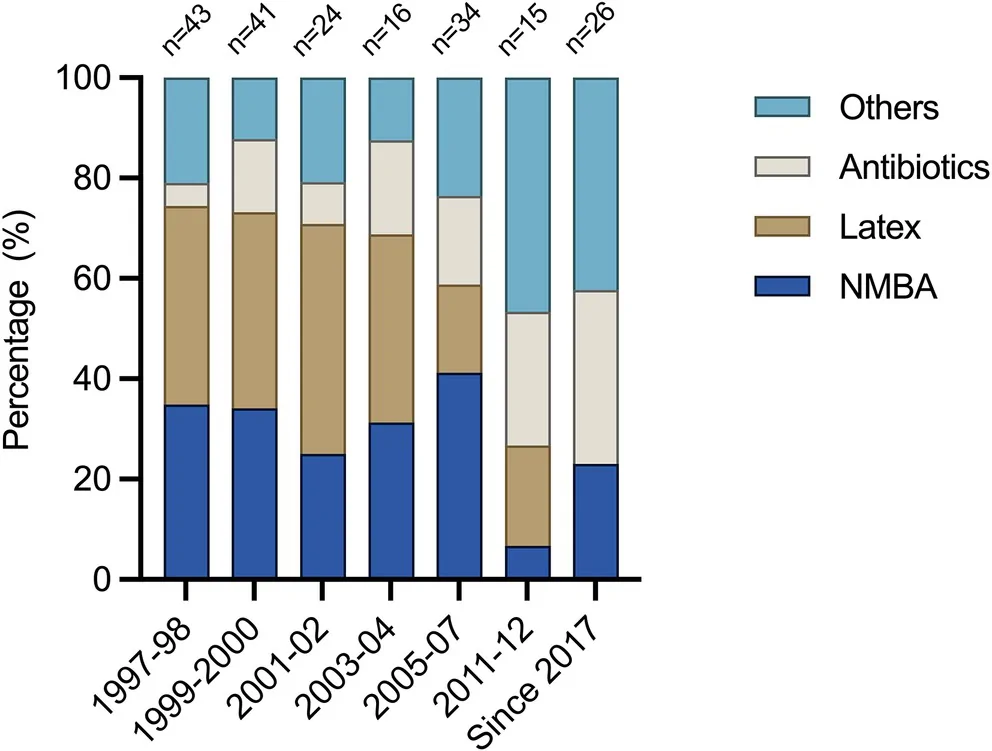
Evolution of the proportion of each identified causal agent according to the year of the GERAP survey in children with POH (1997–2024).

NMBAs and latex were the main causal agents identified, accounting for 61 (31%) and 59 (30%) reactions out of the 199 POH reactions with an identified agent, respectively. The distribution of each category of causal agent significantly changed over time during the study period (*p* = .002), as shown in Figure 2. There was no more latex‐induced reaction after 2017.

**FIGURE 3:**
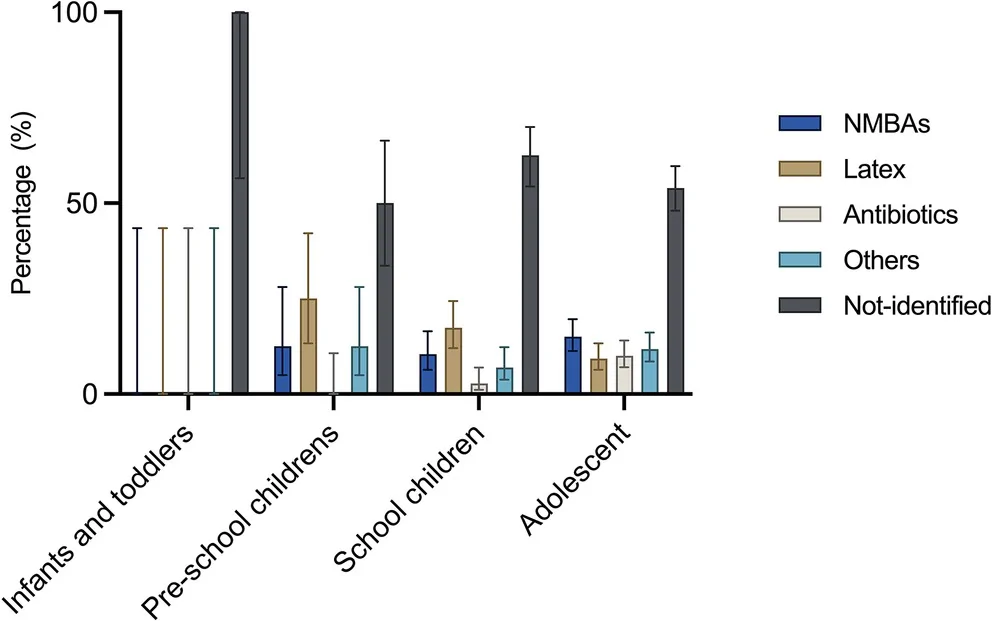
Distribution of the identification of the causal agent according to age categories (infants and toddlers <2 years *n* = 5; pre‐school children 2–5 years *n* = 32; school children 6–12 years *n* = 144; adolescents ≥13 years *n* = 280).

Figure 3 shows the distribution of the identified causal agent by age groups: infants and toddlers (<2 years) (*n* = 5, 1%); pre‐school children (2–5 years) (*n* = 32, 7%); schoolchildren (6–12 years) (*n* = 144, 31%); and adolescents (13–18 years) (*n* = 280, 61%). There was no difference of severity according to the age group (Figure S2).

The distribution of the identified causal agents according to severity is shown in Figure S3. Of the 61 patients with NMBA as the causal agent, 23 (38%) had never been anesthetised before. Patients with NMBA‐induced reactions and no previous exposure to general anaesthesia were mainly schoolchildren and teenagers (*n* = 9 (39%) and *n* = 11 (48%)), respectively. The six grade 4 reactions were attributed to NMBAs in two cases (atracurium and mivacurium), to antibiotics in two cases (cefazolin and one non‐identified antibiotic) and to latex in one case; the remaining case was unexplained despite a complete allergy workup. Acute serum tryptase, available in two of these patients, was markedly raised (51 and 66.5 μg/L).

### 3.4 Patient's phenotypes according to the identification of the causal agent

The causal agent was more frequently identified in children with anaphylaxis compared to children with isolated cutaneous signs (*n* = 141/232 (61%) vs. *n* = 58/229 (25%); *p* < .001). Patients with an identified causal agent more often had severe reactions (grade 3–4: *n* = 92 [46%] vs. *n* = 34 [13%]; *p* < .001) with more frequently cardiovascular (*n* = 86 [43%] vs. n = 52 [20%]; *p* < .001) and respiratory symptoms (*n* = 88 [44%] vs. *n* = 45 [17%]; *p* < .001), with an anaesthetic procedure being more frequently discontinued (*n* = 14 [19%] vs. *n* = 6 [5%]; *p* < .001), compared to patients with an unknown causal agent (Table 3). Median acute tryptase and histamine values were significantly higher in the population of children with a causal agent being identified at 16.0 [5.1–43.0] vs. 4.0 [2.8–8.0] μg/L; *p* < .001 and 25 [5–100] vs. 5 [2–27] nmol/L; *p* = .01, respectively.

**TABLE 3:** Comparison of patients with and without an identification of the causal agent.

Variable	Causal agent identified, *n* = 199	Unknown causal agent, *n* = 262	*p*‐Value
**General characteristics**			
Sex Female	96 (48%)	120 (46%)	0.64
Age (yo)	14 [10–16]	14 [10–16]	0.38
**Medical history**			
Atopy	67 (34%)	78 (30%)	0.42
Asthma	30 (15%)	42 (16%)	0.80
Drug allergy	32 (16%)	34 (13%)	0.42
Food allergy	19 (10%)	16 (6%)	0.21
Latex allergy	31 (16%)	42 (16%)	0.99
Previous POH	4 (2%)	3 (1%)	0.47
Chronic medication with betablockers	1 (1%)	1 (0%)	0.99
Previous anaesthesia	129 (65%)	119 (45%)	**<0.001**
**Type of anaesthesia**			
General anaesthesia	98 (88%)	186 (93%)	**0.02**
General anaesthesia + local regional anaesthesia	4 (4%)	3 (2%)	
Local regional anaesthesia	7 (6%)	2 (1%)	
Local anaesthesia	2 (2%)	9 (5%)	
**Timing of the reaction**			
Induction	58 (61%)	126 (69%)	0.13
Maintenance	23 (24%)	26 (14%)	
Recovery	14 (15%)	30 (17%)	
**Description of the reaction**			
Cutaneous signs	153 (77%)	223 (85%)	**0.03**
Cardiovascular signs	86 (43%)	52 (20%)	**<0.001**
Respiratory signs	88 (44%)	45 (17%)	**<0.001**
**Severity grade**			
Grade 1	58 (29%)	171 (65%)	**<0.001**
Grade 2	49 (25%)	57 (22%)	
Grade 3	87 (44%)	33 (13%)	
Grade 4	5 (3%)	1 (0%)	
**Decision after the reaction**			
Continued	60 (81%)	112 (95%)	**<0.001**
Reported	14 (19%)	6 (5%)	
**Allergy workup**			
Acute serum tryptase (μg.L^−1^)	16.0 [5.1–43.0]	4.0 [2.8–8.0]	**<0.001**
Acute histamine (nmol.L^−1^)	25 [5–100]	5 [2–27]	**0.01**
Time interval between reaction and allergy workup, months	3 [2–8]	4 [2–9]	0.11

*Note*: Data are presented as *n* (%) and median [IQR].

Abbreviation: POH, perioperative hypersensitivity reaction.

## 4 DISCUSSION

In this study, we describe the largest cohort of paediatric POH, detailing causal agents, severity, temporal trends, age‐specific peculiarities and phenotypes according to identification of a causal agent.

Paediatric POH accounted for 10% of all POH cases in our database. This proportion is already substantial and may even be underestimated, given the tendency to underestimate the risk of POH in the paediatric population. Similarities with adults are common, for example, most reactions occurred during induction of general anaesthesia.^1^ However, in contrast to adults, half of the paediatric cases were grade 1 reactions, and the causal agent was identified in only 25% of these patients. In the specific subpopulation of paediatric patients with anaphylaxis, the identification rate increased to 61%. This difference raises the question of whether Grade 1 reactions and anaphylaxis share the same underlying mechanisms ‐ a question that skin testing, BAT or mast cell activation test could help answer. In fact, the causal agent was identified in only 43% of paediatric patients, which is lower than the rate observed in adults, where the causal agent is identified in 60 to 70% of cases.^3^ Despite their age, the vast majority of our cohort had skin test results, indicating that these patients had been truly investigated. As Green et al. explained, skin testing in children may be difficult due to their fear of being stung.^19^ The cooperation of the young patient and their parents is essential for high‐quality skin tests, especially intradermal tests. Testing may sometimes need to be delayed until the child is old enough to understand and consent, even though the delay may then yield false‐negative results. This was however not the case in our study, in which patients in whom the causal agent was unknown were evaluated at the same time interval as those in whom the causal agent was identified. Our data confirm the feasibility of skin testing in children, even young ones, with the youngest patients at 2 years of age, which should encourage allergists to evaluate these patients early within the POH period to limit the risk of false‐negative results due to delayed evaluation. As shown in Table 3, patients with an identified causal agent had higher acute tryptase and histamine levels, consistent with mast cell activation. The mechanisms underlying POH in children remain incompletely understood and may differ between phenotypes; some reactions in our cohort, particularly the milder ones, may reflect non‐specific mechanisms, whereas the higher identification rate observed in more severe reactions is compatible with IgE‐mediated mechanism, possibly involving immunoglobulin maturation, although this hypothesis requires confirmation. In adults, guidelines recommend investigating all patients with POH, including blood samples collected during the reaction and skin testing 4–6 weeks after the reaction. The same is true in children: all patients with a suspected POH need to be referred to an allergist and have blood samples during the reaction to better assess the mechanism of the reaction. Although a 25% identification rate for grade I reactions may seem low, these patients are at real risk of recurrence ‐ even more severe‐ on re‐exposure and must be fully evaluated. The low rate of biological sampling in our cohort is striking and suggests that targeted education towards anaesthetists may be useful in this population.

Our results showed that NMBAs, latex and antibiotics were the main causal agents of POH in France. As shown in Figure 2, latex was the main causal agent from surveys 5 to 8, before the general implementation of primary prevention measures, such as the complete removal of latex from paediatric hospitals and the use of unpowdered gloves, and secondary prevention measures, such as better identification of patients with latex allergies. After the eighth survey, the number of latex‐related reactions started to decrease and finally completely disappeared after 2017. This shows that preventive measures, especially the exclusion of latex from children's hospitals, can effectively control the risk of allergy if the risk is properly identified. The number of reactions triggered by NMBA varies greatly between studies, but remains quite important until the 9th survey. After that, antibiotic‐related reactions seem to increase, although we were unable to determine whether this observation is clinically relevant. Interestingly, NMBA‐related reactions may occur in very young patients (aged 2–5 years), while antibiotic‐related reactions occur later, after the age of 5. While previous exposure to antibiotics may explain why these reactions occurred after a certain age, the occurrence of NMBA‐related reactions in patients with no history of general anaesthesia raises the question of how they were sensitised. In France, the ALPHO case–control study showed that consuming pholcodine in the year before anaesthesia involving NMBA increased the risk of an NMBA‐related reaction by 4.2.^20^ In response, the European Medicines Agency removed pholcodine from the European market in 2023. Therefore, it is possible that some of these patients had taken pholcodine. Other environmental factors may also contribute to sensitisation to NMBA, such as quaternary ammonium compounds present in cleaning products or shampoo. However, we were unable to determine whether our patients were exposed to these products or not.

Chlorhexidine was identified in only one case in our cohort, in contrast to some countries where chlorhexidine‐related reactions account for approximately 9% to 10% of perioperative anaphylaxis. In the most recent GERAP survey in 2017–18, a period in which chlorhexidine hypersensitivity was systematically assessed, only 4 (1%) patients were found to be positive.^1^ Therefore, the single paediatric case in our cohort reflects truly low exposure rather than incomplete testing.

Only few studies described the results of allergy workup in the paediatric population, with identification of the causal agent largely distributed from 38% to 72% (close to the adults' rate). Among 15 POH cases, Toh et al. reported the identification of the causal agent in 5 cases (of 8 fully investigated).^7^ Over the 11 cases included in the NAP6 study, the causal agent was identified in 8 cases (72%).^4^ Over 29 cases of paediatric POH, Aydemir et al. described the identification of the causal agent in 11 patients (38%), mainly rocuronium, chlorhexidine and latex.^9^ In a Danish cohort of 70 patients, Madsen et al. reported the identification of the causal agent in 24% of cases, mainly chlorhexidine, NMBAs and antibiotics.^21^ In their cohort of 27 patients with POH, Green et al. identified cefazolin as the main causal agent, accounting for 17 cases (63%). NMBAs, on the other hand, accounted for only seven cases.^19^

Females typically predominate in adult POH cohorts, accounting for approximately two‐thirds of all cases. Females represented only 47% of our paediatric POH cohort: males were more prevalent among school‐aged children, while females were more prevalent during their teenage years. This observation is consistent with previous findings that females become more prevalent after puberty, suggesting that hormonal factors play an important role in the process of drug sensitisation. Males and females are not equally exposed to surgery, and therefore anaesthesia, during childhood. In the French APRICOT data, females represented only 39% of the cohort.^22^ Therefore, boys are more likely to be exposed to anaesthesia during childhood, which may explain the overrepresentation observed in school‐aged children, particularly those exposed to trauma. Similarly, the relative balance observed in adolescents may be influenced by the underrepresentation of females in this age group during surgery.

Our study had several limitations. The main limitation arose from its retrospective nature, with some missing data precluding a more detailed analysis of paediatric POH. Some data were not recorded in earlier GERAP surveys and could therefore not be used in our analysis. The absence of basal serum tryptase assessment in the vast majority of our cohort meant that it was impossible to study the clinical relevance of formula intake in relation to acute and basal tryptase levels in order to characterise mast cell activation in our population. Data on the subsequent anaesthetic management of these patients and on possible recurrence on re‐exposure were not recorded in the database and therefore could not be analysed. Due to the retrospective nature of this study, we were also unable to strictly control the allergy workup conducted in every patient, even though all GERAP centres comply with the most recent guidelines, and French guidelines have long recommended strict adherence to non‐irritating concentrations. The scope of testing may have differed between centres, with some applying a broad panel of general anaesthetics and others testing only the clinically suspected drugs. As this study covers nearly 30 years of surveys, BAT results were not available, and BAT are not available in all GERAP centres. Because the GERAP is a national network of allergy centres based on spontaneous referral rather than a population‐based registry with a defined catchment area, the total number of paediatric anaesthetic procedures performed in the contributing centres is not available. A France‐specific incidence of paediatric POH could therefore not be calculated, and only the proportion of paediatric cases among all recorded POH (10%) can be reported.

In conclusion, based on the largest cohort of paediatric cases of POH ever published, our results showed that, while paediatric cases of POH are not uncommon, they are generally less severe than in adults. However, anaphylaxis and near‐fatal events in the perioperative setting may also occur in children. Considering that proven POH with identified causal agent are more severe and potentially life‐threatening compared to other hypersensitivity reactions, our results highlight the crucial need to explore all suspected POH in children in specialised allergy centres for a better prevention of further general anaesthetic procedures. Current prevention measures appear to control the risk of latex‐related reactions, but NMBA and antibiotics remain significant causal agents involved in these reactions. The mechanisms behind the sensitisation of young patients to NMBA remain unclear and further investigation is needed to identify the environmental factors responsible for this process.

## AUTHOR CONTRIBUTIONS

**Karila Chantal:** Writing – review and editing; investigation. **Pouessel Guillaume:** Writing – review and editing; investigation. **Mertes Paul Michel:** Conceptualization; writing – review and editing; methodology; supervision. **Franchina Sébastien:** Writing – review and editing; investigation. **Lejus‐Bourdeau Corinne:** Writing – review and editing; investigation. **Gouel Aurélie:** Writing – review and editing; investigation. **Tacquard Charles:** Conceptualization; data curation; formal analysis; writing – original draft; writing – review and editing; methodology; investigation.

## FUNDING INFORMATION

The authors have nothing to report.

## CONFLICT OF INTEREST STATEMENT

P.G. has provided consultation and speaker services for AImmune Therapeutics, Stallergenes, Novartis, DVB technology, ALK‐Abello, Bioprojet; serves as a medical consultant/advisor for Bioprojet, ALK‐Abello. The other authors declare no conflicts of interest.

## Supporting information


**Figure S1.** Distribution of cases by age and gender. Infants and toddlers <2 years; pre‐school children 2–5 years; school children 6–12 years; adolescents ≥13 years. Results are expressed as percentage and 95% confidence interval.
**Figure S2:** Distribution of the severity grade in each age group. Data are expressed as percentage and 95% confidence interval.
**Figure S3:** Distribution of identified causal agents according to the severity of the reaction.
**Table S1:** Data availability according to the source.
**Table S2:** Diagnostic method(s) used to identify each identified causative agent of paediatric perioperative hypersensitivity (*n* = 199).

## Data Availability

The data that support the findings of this study are available from the corresponding author, T.C., upon reasonable request.
