# Supplementary material for: Paediatric perioperative hypersensitivity reactions: Analysis of the French GERAP network database
Source: Pediatr Allergy Immunol. 2026 Sep 16;37(9):e70474. doi: 10.1111/pai.70474 (PMC13580975; doi:10.1111/pai.70474)
Supplement: Supplementary file 1 [file PAI-37-e70474-s001.docx]

**Paediatric perioperative hypersensitivity reactions: analysis of the French GERAP network database**

Short title: Paediatric perioperative hypersensitivity reactions

**Mertes Paul Michel,** M.D., Ph.D. UMR_S1255 EFS-INSERM unit, EFS Grand Est, Strasbourg, France

**Pouessel Guillaume**, M.D. Department of Paediatrics, Children’s Hospital, Roubaix, France;

Paediatric Pulmonology and Allergy Department, Jeanne de Flandre Hospital, Lille University Hospital (CHU), Lille, France; Lille University, ULR 2694: METRICS, F-59000, Lille, France;

**Gouel Aurélie**, M.D., Ph.D. University Paris Cité, Paris, France**,** Anesthesiology and Critical Care Medicine Department, DMU PARABOL, Bichat Hospital, AP-HP, Paris, France **;** Antibody in Therapy and Pathology, Pasteur Institute, UMR 1222 INSERM, Paris, France

**Karila Chantal**, M.D., Paediatric Pulmonology and Allergy Department, Necker-Enfants malades Hospital, Paris-Descartes University, 75015 Paris, France

**Lejus-Bourdeau Corinne**, M.D., Ph.D., Department of anaesthesia and intensive care, CHU Nantes, Hôtel Dieu - Hôpital Mère Enfant, F-44000 Nantes, France

**Franchina Sébastien**, M.D. Perioperative Allergic Reaction Exploration Unit, Department of Anesthesia and Intensive Care, Rouen University Hospital, Rouen, France

**Tacquard Charles**, M.D., Ph.D., Department of anaesthesia and intensive care, Strasbourg University hospital, Strasbourg, France; UMR_S1255 EFS-INSERM unit, EFS Grand Est, Strasbourg, France

**For the GERAP Network**

**Supplementary Figure 1:** Distribution of cases by age and gender. Infants and toddlers < 2 years; pre-school children 2-5 years; school children 6-12 years; adolescents ≥ 13 years. Results are expressed as percentage and 95% confidence interval.

**Supplementary Figure 2**: Distribution of the severity grade in each age group. Data are expressed as percentage and 95% confidence interval.

**Supplementary Figure 3:** Distribution of identified causal agents according to the severity of the reaction.

|  | **Survey N°5** (1997-98)  n=44 | **Survey n°6** (1999-2000)  n=99 | **Survey N°7** (2001-02)  n=67 | **Survey n°8** (2003-04)  n=56 | **Survey n°9** (2005-07)  n=100 | **Survey n°10** (2011-12)  n=39 | **Data after 2017**  n=56 |
| --- | --- | --- | --- | --- | --- | --- | --- |
| Age/sex | X | X | X | X | X | X | X |
| Medical history | X | X | X | X | X | X | X |
| Type of anaesthesia |  | X | X | X | X | X | X |
| Anaesthetic protocol |  | X | X | X | X | X | X |
| Severity grade | X | X | X | X | X | X | X |
| Clinical signs | X | X | X | X | X | X | X |
| Timing of the reaction |  | X | X | X | X | X | X |
| Decision for the surgery |  |  | X | X | X | X | X |
| Time interval between reaction and workup |  | X | X | X | X | X | X |
| Serum histamine value (acute) |  |  |  |  | X | X | X |
| Serum tryptase (acute) |  | X | X | X | X | X | X |
| Serum tryptase (baseline) |  |  |  |  |  |  | X |
| Specific IgE | X | X | X | X | X | X | X |
| Skin tests | X | X | X | X | X | X | X |
| Challenge tests | X | X | X | X | X | X | X |
| Conclusion | X | X | X | X | X | X | X |

**Supplementary table 1**: Data availability according to the source.

| Causal agent | Identified cases, n | Positive skin tests | Positive specific IgE | Positive drug provocation |
| --- | --- | --- | --- | --- |
| Neuromuscular blocking agents | 61 | 60 | 22 | 0 |
| Latex | 59 | 55 | 47 | 0 |
| Antibiotics | 31 | 30 | 3 | 1 |
| Hypnotics | 14 | 14 | 0 | 0 |
| Opioids | 8 | 8 | 0 | 0 |
| Colloids | 5 | 5 | 0 | 0 |
| Local anaesthetics | 2 | 2 | 0 | 0 |
| Other | 19 | 17 | 0 | 2 |
| Total | **199** | **191** | **72** | **3** |

**Supplementary Table 2.** Diagnostic method(s) used to identify each identified causative agent of pediatric perioperative hypersensitivity (n = 199).
